# Developing dendritic cell for SARS-CoV-2 vaccine: Breakthrough in the pandemic

**DOI:** 10.3389/fimmu.2022.989685

**Published:** 2022-09-06

**Authors:** Jonny Jonny, Terawan Agus Putranto, Raoulian Irfon, Enda Cindylosa Sitepu

**Affiliations:** Cellcure Center, Gatot Soebroto Central Army Hospital, Jakarta, Indonesia

**Keywords:** dendritic cells, immunotherapy, T cells, SARS-CoV-2, vaccine candidate, vaccine approach

## Abstract

Finding a vaccine that can last a long time and effective against viruses with high mutation rates such as SARS-CoV-2 is still a challenge today. The various vaccines that have been available have decreased in effectiveness and require booster administration. As the professional antigen presenting cell, Dendritic Cells can also activate the immune system, especially T cells. This ability makes dendritic cells have been developed as vaccines for some types of diseases. In SARS-CoV-2 infection, T cells play a vital role in eliminating the virus, and their presence can be detected in the long term. Hence, this condition shows that the formation of T cell immunity is essential to prevent and control the course of the disease. The construction of vaccines oriented to induce strong T cells response can be formed by utilizing dendritic cells. In this article, we discuss and illustrate the role of dendritic cells and T cells in the pathogenesis of SARS-CoV-2 infection and summarizing the crucial role of dendritic cells in the formation of T cell immunity. We arrange the basis concept of developing dendritic cells for SARS-CoV-2 vaccines. A dendritic cell-based vaccine for SARS-CoV-2 has the potential to be an effective vaccine that solves existing problems.

## Introduction

COVID-19, which WHO declared a pandemic in March 2020, remains the focus of world problems (1). The infection is caused by the SARS-CoV-2 virus, a positive-strain RNA virus that belongs to the beta coronavirus family *(* 2 *).* SARS-CoV-2 conveys a genome resemblance to the MERS-CoV and SARS-CoV viruses (3). SARS-Cov-2 continues to mutate, giving rise to various variants of this virus. Some emerging variants classified as Variance of Concern (VoC) include the alpha, beta, delta, and omicron variants (4).

The SARS-CoV-2 infection manifests into various organ system abnormalities such as the respiration, cardiovascular, nervous, and digestive systems with a broad spectrum of symptoms ranging from mild to severe (5). In SARS-CoV-2 infection, various pathology findings were documented, such as a decrease in the number of lymphocytes to an increase in inflammatory cytokines production that led to cytokine storm in severe symptomatic patients (6). These findings indicate the failure of human immune response in SARS-CoV-2 infection. The immune system failure is attributed to the ability of SARS-CoV-2 to evade the human immune response. Specifically, T cell dysfunction was found in SARS-CoV-2 infection, which is essential in eliminating SARS-CoV-2 in the body (7).

To date, various types of vaccines have been developed and approved to prevent SARS-CoV-2 infection. All of these vaccines are oriented to produce antibodies that can neutralize SARS-CoV-2. However, studies show that there is a decline in antibodies several months after vaccination and also a decrease in the effectiveness of existing vaccines against the evolving variants of SARS-CoV-2 (8). This has implications for the need of the novel effective vaccine development to protect against the emergence of SARS-CoV-2 variants. Meanwhile, it has been known that memory T cells are capable of lasting longer than the antibodies formed and have the capability to recognize the SARS-CoV-2 variants (9). Therefore, the development of a T cell-oriented vaccine is a promising approach for the generation of effective and long-lasting immunity against SARS-CoV-2.

Dendritic cells (DC) have a pivotal role in the immune system, which connects the activation of the innate and adaptive immune systems. In addition, DC is well-known for its ability to activate and differentiate naïve T cells (10). DC has been developed as an immunotherapy or vaccine for cancer and infections (11). DC’s ability to activate the immune system, the successful development of DC-based immunotherapy in other diseases, and also considering the role of DC in the COVID-19 can be the cornerstone for the development of DC-based vaccine for SARS-CoV-2. Therefore, this article discuss the potential development of DC as a SARS-CoV-2 vaccine by focusing on the role of T cells and DC in SARS-CoV-2 infection, the formation of immunity in SARS-CoV-2 infection, and the role of DC in shaping immunity which is the foundation for the development of DC as a SARS-CoV-2 vaccine.

## Immune system dysfunction in SARS-CoV-2 infection

Viruses that invade the body first will activate an innate immune response that aims to eliminate the virus and then trigger an adaptive immune response. RNA Viruses such as SARS-CoV-2 have Pathogens Associated Molecular Patterns (PAMPs) that can be recognized and bonded to Patterns Recognition Receptors (PRR) in the cytosol and endosomal phagocytic cell (12). This process leads to polynuclear lymphocyte cells, monocytes, Natural Killer (NK) cells along with DC recruitment (13). Recruitment of these cells is a crucial process that intends to eliminate the virus and stop the disease progression. Antigen Presenting Cell (APC) captures incoming viral particles to be introduced to naïve T cells (14). Naïve T cells then differentiate into specific CD4+ and CD8+ T cells (15). There are two kinds of CD8+ T cells: effector T cells or cytotoxic T cells (Tc) and memory cells. These formed Tc cells are responsible for eliminating the virus. CD4+ T cells or T helper (Th) assist the role of Tc and contribute to the formation of the humoral immune system by differentiating B cells into B cell-producing specific antibodies (16).

There are several immunopathologies found in COVID-19. Studies revealed the presence of lymphopenia and increased activation of T cells, which are the characteristics of lymphocyte dysfunction, abnormalities in monocytes and granulocytes, increased cytokines production, and the generation of specific antibodies, especially in patients with severe symptoms (17, 18). All these hallmarks correlate to severity degree and survival rate (19). These conditions also indicate the presence of both innate and adaptive immune dysfunctions by which the SARS-CoV-2 capability to evade the immune responses (20).

The invading SARS-CoV-2 will be identified by Retinoid-acid Inducible Gene-1 (RIG-1), Melanoma-Differentiation Associated protein 5 (MDA-5), Toll-like Receptor 7 (TLR-7), and TLR-4 which specifically recognize S SARS-CoV-2 glycoprotein (21). The process activates the transcription of Nuclear Factor kappa-B (NF-kB), Interferon Regulatory Factor 3 (IRF-3), and IRF-7 (22). Under normal circumstances, the invading virus initiates the provision of type I interferon (IFN-I), IFN-III, pro-inflammatory cytokines, in conjunction with chemokines (6). At the early phase of the disease, IFN-I plays a critical role in eliminating and inhibiting viral replication and assisting in activating adaptive immune responses (23). However, delays in the provision and activity of IFN-I will trigger the progressivity of SARS-CoV-2 infection (24). In SARS-CoV-2 infection, there was a suppression and delay in the IFN-I provision (25). It is caused by inhibition of signaling pathways by Open Reading Frame 3b (ORF3b), ORF4a, ORF4b, ORF5, ORF6, Non-specific protein 1 (Nsp1), Nsp2, Nsp14, M, and N SARS-CoV-2 (21). The suppression of IFN-I is a mechanism by which SARS-CoV-2 avoids the immune system that leads to unrestrainable viral replication and disease progressivity (26).

**Figure 1:**
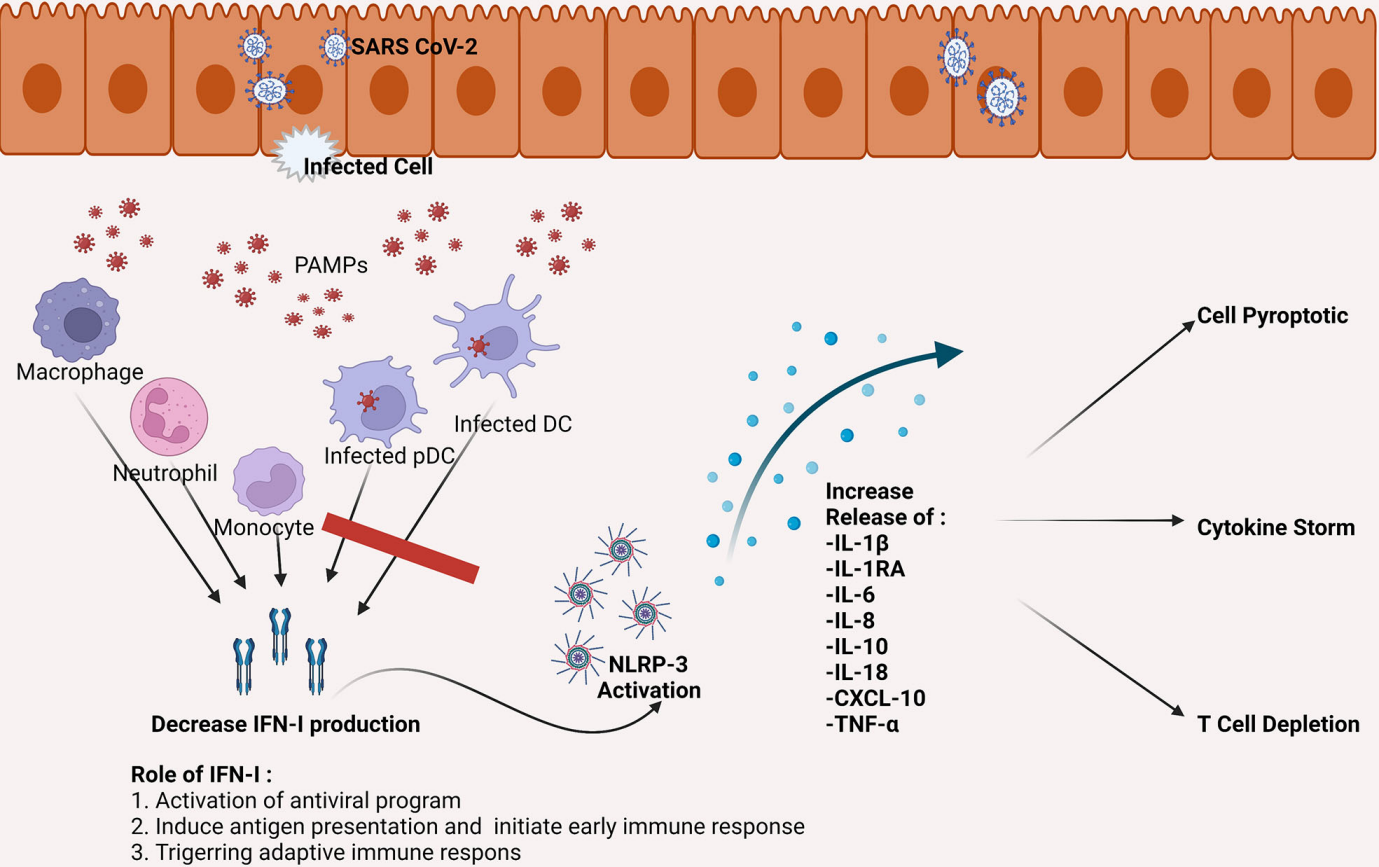
SARS-CoV-2 can infect DC, including pDC, which is the primary producer of IFN-I. The SARS-CoV-2 infection causes a decrease in the number of DC as well as a decrease in IFN-I production. Inadequate IFN-I leads to failed elimination of SARS-CoV-2. The failure eventually increased the activity of NLRP-3, which leads to pro-inflammatory cytokines increase which then triggers cell apoptotic, cytokine storms, and depletion of T cells. CXCL, the chemokine C-X-C motif ligand; DC, dendritic cell; IFN, interferon; IL, interleukin; NLRP-3, NLR family pyrin domain containing 3 inflammasome; pDC, plasmacytoid dendritic cell; TNF, tumor necrosis factor.

Failure to eliminate SARS-CoV-2 leads to an increase in activation of Nod-like Receptor Family Pyrin Domain Containing 3 (NLRP3) inflammasome (27). This condition contributes to severe inflammatory reactions and severe progressivity of the disease. In COVID-19, NLRP3 activation involves the appearance of programmatic cell death through the production of interleukin 1β (IL-1β) and IL-18, which induces leucopenia (28). NLRP3 activation also increases macrophage activation, thus, increasing the production of IL-1RA, IL-6, IL-8, IL-10, Tumor Necrosis Factor-Alpha (TNF-α), and chemokine C-X-C ligand 10 (CXCL-10) (29). This process is one of the factions of the occurrence of cytokine storms in COVID-19 patients (see **Figure 1**) (30).

Cellular adaptive immune responses play an important role in the pathogenesis of COVID-19, which involves SARS-CoV-2-specific CD4+ and CD8+ T cell activity (31). T cells will respond to SARS-CoV-2 through the recognition of the SARS-CoV-2 epitope presented by MHC (32). The main targets of T cells are the M, N, S, and other various epitope proteins expressed by ORF3, ORF8, Nsp2, and Nsp4 SARS-CoV-2 (33). Approximately, there are 1.400 SARS-CoV-2 epitopes recognizable by T cells (34). Studies have shown that most epitopes are retained in various variants of SARS-CoV-2 (35).

Earlier induction of CD8+ T cell was found in the patients with mild symptoms (36). This demonstrates the critical role of CD8+ T cells in eliminating the SARS-CoV-2. In the severe patients, there was an escalation in T cells activation, especially CD8+ T, which was characterized by an increase in the expression of several activation markers (CD38, Human Leukocyte Antigen-DR isotype/HLA-DR, Ki-67) and cytotoxic proteins (perforin and granzyme B) (37). T cells activation leads to the T cells fatigue. This condition is characterized by increased inhibitor receptors expression such as Lymphocyte Activation Gene 3 (LAG-3), T-cell Immunoglobulin and Mucin Domain-Containing Protein 3 (TIM-3), and also Programmed Cell Death Protein-1 (PD-1) (37, 38). The fatigue T cells will have a reduction in their cytotoxic ability thus, they are ineffective in eliminating the virus.

There were CD4+ and CD8+ T cell numbers declining peculiarly in severe patients, indicating the presence of T cell dysfunction in COVID-19 infection (39). Several mechanisms have been thought to cause the decrease in the T cell counts. First, it is caused by viral infection directly through the ACE receptors owned by T cells (35). Second, it is caused by the suppression of the infected lymphoid organs so that there is a decrease in lymphocyte production (40). Third, it is caused by the process of T cell apoptosis mediated by the bond of Fas and Fas Ligand (FasL). In COVID-19, Fas expression on the surface of T cells and plasma FasL production was found to increase (41). Fourth, the presence of T cell pyroptotic induced by the upregulation of NLRP-3 (29). Fifth, direct cytopathic effects on T cells by IL-6 and TNF-α (42). Sixth, T cell apoptosis mediated by infected DC, characterized by an increase in Tumor Necrosis Factor-related Apoptotic Inducing Ligand (TRAIL) in the DC (43).

SARS-CoV-2 has been shown to have the ability to infect DC, causing a decrease in the DC’s number and DC’s function impairment. SARS-CoV-2 infection can reduce the number of mononuclear DC (moDC) by 10-20% (44). Studies in COVID-19 patients in acute and convalescent-phase showed a decrease in the conventional DC (cDC) and plasmacytoid (pDC) number accompanied by an increase in the cDC/pDC ratio, especially in patients with severe symptoms (45). There was also a pDC decrease in pediatric patients who experienced Multisystem Inflammatory Syndrome in Children (MIS-C) due to SARS-CoV-2 infection (46). Depletion in cDC and pDC number remained found until seven months post-infection (47).

SARS-CoV-2 infection also causes DC maturity impairment. Examination of patient alveolus tissue showed an increase in DC recruitment that did not have maturity molecules (48). Studies showed a decrease in Human Leucocyte Antigen – DR isotype (HLA-DR) and CD80 expressions, which are the markers of DC maturity, and a reduction in STAT2 activity, which correlates with correlates to the ability of DC to activate CD8+ T cells (43, 49, 50). The immature DC is unable to present antigens to T cells, so the differentiation and production of specific T cells are inadequate (51).

The decrease and dysfunction of DC caused by SARS-CoV-2 infection results in an IFN-I reduction. SARS-CoV-2 inhibits the phosphorylation of STAT1 in moDC and pDC, which leads to suppression and delaying the production of IFN-I (44). The infected DC also produced pro-inflammatory cytokines (IL-6, TNF-α) as well as chemokines (Interferon gamma-induced Protein 10/IP-10, Macrophage Inflammatory Protein 1 alpha/MIP-1α, Monocyte Chemoattractant Protein1/MCP-1 (see **Figure 1**) (51). Thus, SARS-CoV-2 infection in DC has responsible for immune system dysfunction.

## Specific immunity against SARS-CoV-2

Antibodies will be formed when SARS-CoV-2 infection occurs. Immunoglobulin M (IgM) and IgG that are specific to the N and S protein begin to be measured on day 2 of symptoms. IgM peaks on day 11-13 then decrease after 3 weeks besides IgG will be observed entirely on day 17-19 (52). The increase in IgG is followed by the formation of memory B cells for up to 3 months in length (53). Nevertheless, some patients with mild or asymptomatic symptoms were not found to be any seroconversion of these antibodies (54). Studies have also shown a decrease in these antibodies in the 3-6 months (55). Tiandan et al. found that the IgG ability to neutralize SARS-CoV-2 in 1-year post-onset was only 43% subjects, and its antibody ability would decrease against new variants of SARS-CoV-2 (56).

SARS-CoV-2 infection also forms a T-cell response (57). The CD4+ T-cell response was detected in all patients, while CD8+ T cells were found in most patients, not in all patients (33). CD8+ T-cells can be observed on day seven and peak until day 14 (58). T cell responses also remained to be found in mild or asymptomatic patients, despite absent antibodies seroconversion (54). The detected T cell response was characterized by the formation of effector and memory T cells. The formed memory T cells are capable of recognizing various epitopes of SARS-CoV-2 (59). formation of specific memory T cells forms immunity and prevention against reinfection. This finding indicates the superiority of T cell immunity compared to antibodies in preventing the infection.

The memory CD8+ T cells were found to be diverse, ranging from central memory (Trm), effector memory (Tem), resident memory (Trm), even into polyfunctional memory cells or memory T cells that can act as *stem cells* (Tscm) (60). The ability of memory CD8+ T cell formation is attributed to the recognition and elimination ability of SARS-CoV-2 (61). Transient T cell formation CD4+ memory is correlated with the presence of B cells and the production of IgG (53). The specific T cells remain observed for up to 6 months post-infection (62). While polyfunctional T cells remain detected for up to 10–12 months (60). This suggests that SARS-CoV-2 specific T cells can persist for an extended period. This condition shows similarities to SARS-CoV infection in which specific memory T cells remain detected for 17 years (63).

Currently, various vaccines have been developed and used to strengthen immunity against SARS-CoV-2. There are several types of vaccines in circulation, such as protein-based vaccines, messenger ribonucleid acid (mRNA), viral vectors, and inactivated viruses (8). All types of vaccines have the formation of specific antibodies that can neutralize SARS-CoV-2 with varying efficacy. mRNA-based vaccines show effectiveness above 90% (64, 65), virus vector-based vaccines 66-91% (66, 67), inactivated virus-based vaccines can reach 80% (68), while protein-based vaccines are currently still being developed (69). However, research shows a decrease in the effectiveness of all these vaccines against VoC by 0.5–11 times (8).

## Role of dendritic cell in shapingT cell immunity

DC is well-known as the most potent APC and plays a pivotal role in innate and adaptive human immune systems (10). In the innate immune system, DC introduces and determines the body’s response to DAMP or PAMP. In the adaptive immune system, DC is responsible for presenting antigens to naïve T cells (70). DC exposed to the antigen will maturate and drain to the lymphoid organs, then present the antigen to the naïve T cells leading to T cell differentiation (71). Therefore, DC has a role in connecting the innate and the adaptive immune system.

DC is derived from Lymphoid Primed Multi-Potent Progenitor (LMPP) which differentiates into Granulocyte-Macrophage DC progenitor (GMDP) and then becomes macrophage DC progenitor (MDP). MDP will be a Common DC Progenitor (CDP) that will differentiate into pDC, cDC1, and cDC2 (72). In addition, there is DC derived from monocytes (moDC) and DC subset known as Langerhans cells (10). In general, there are five types of DC. pDC, cDC1, and cDC2 are DC found under any conditions, while Langerhans cells are specified in the skin, while moDC is only produced when there is inflammation. DC can be found in the lymphoid organs, circulation, and specific tissues or organs such as the lungs, liver, and digestive tract (73).

**Figure 2:**
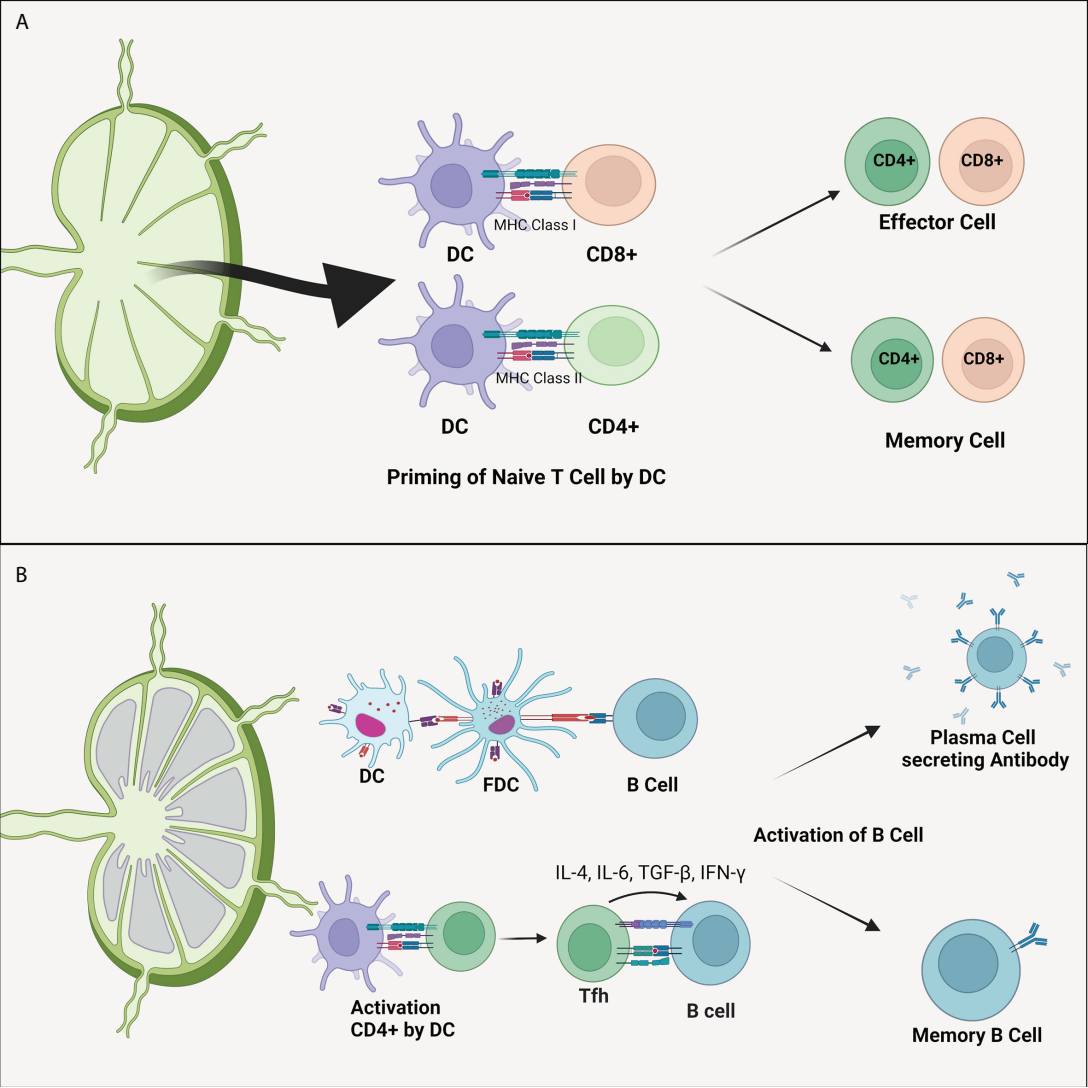
**(A)** Dendritic cell’s ability to differentiate naïve T cells. As APC, DC presents antigens to CD4+ and CD8+ T cells through MHC-II and MHC-I, respectively. This process forms antigen-specified effector and memory T cells. **(B)** Dendritic cell also plays a vital role in the B cells activation. Activation of B cells can be directly carried out by DC cells or by the intermediately by CD4+ T cells. Activated CD4+ T cells will migrate to the follicular area to activate specific B cells of both plasma B cells that produce antibody and memory B cells. DC, dendritic cell; FDC, follicular dendritic cell; IL, interleukin; MHC, major histocompatibility complex; TGF-β, transforming growth factor β.

The critical role of DC in the immune system is to perform priming cell T (**Figure 2**). This process differentiates naive T cells into antigens or pathogen-specific T cells (10). Memory T cells will cause pathogen elimination to occur faster and prepare the body for repeated pathogens exposure (74). DC presents antigens to CD4+ through MHC-II molecules and CD8+ *via* MHC-I (75). Activation of CD4+ T cells by DC will induce the formation of plasma cells so that specific antibodies are formed (**Figure 2B**). In addition to the ability to recognize external antigens, DC can also recognize self-antigens in the body to prevent the occurrence of autoimmune through priming T cell becomes cell T regulator (Treg) (71). T cells priming process is affected by the presence of antigen presentations, co-stimulating molecules, and the presence of cytokine production (70).

Each type of DC has its function (**Table 1**). pDC can be found in the circulation and lymphoid organs and plays a crucial role in the body’s immune mechanism against viruses because it has TLR that can recognize RNA and DNA (81). Besides as primary producer of IFN-I (such as IFN-α), pDC also produces IFN-III, TNF-α, IL-6, and granzyme B *(* 72 *).* CD4+ T cells can be primed by pDC by CD-303 and CD-367 molecules, while CD8+ T cells are primed by pDC through antigen transfer to cDC and the resulting IFN-I activity (76).

**Table 1:** Types of dendritic cells and their functions.

Dendritic Cell Types	Function	References
**Plasmacytoid Dendritic Cell (pDC)**	Priming of CD8+ through IFN-I production and antigen transfer to cDC and priming CD4+ through regulation of CD-303 and CD-367 molecules	(72, 76)
**Conventional Dendritic Cell 1 (cDC1)**	Regulate and prime CD8+ by IFN-III, CXCL 9/10, and IL-12 production	(77)
	Priming Trm by the production of CD-24, IL-12, IL-15	(70)
	Differentiation Th1 and Tfh that induced of B cell	(16, 72)
**Conventional Dendritic Cell 2(cDC2)**	Potent activator of Th1, Th2, Th17 through IL-1β, IL-6, IL-12, dan IL-23 production	(73)
	Differentiates CD8+ and regulating Tcf1	(78)
	The efficient inductor of Tfh	(16)
	Differentiates Treg through the production of IL-10 and TGF-β	(72)
**Monocyte derived Dendritic Cell (moDC)**	CD4+ and CD8+ T cells priming through regulation of Tbet, Tcf1 and by producing cytokines in inflammation states.	(72, 79)
	Differentiates long term memory T cells by producing IL-15	(80)
**Langerhans Cell**	Specific immune responses in the skin	(10)

Conventional dendritic cells 1 (cDC1) are more prevalent in tissues than blood (73). cDC1 activates effector CD8+ T cells and NK cells through the C-X-C chemokine Ligand motif 9 (CXCL9), CXCL10, and XC 1 chemokine receptors (CXR1) expression so that it can regulate cytotoxic cells (77). In addition, cDC1 can also activate Trm through CD-24 expression and the production of IL-12 and IL-15 (70). These cytokine productions can also activate Th1 cells (72). Studies show that cDC1 also plays a role in the activation of Tfh. In addition, Th1 and Tfh produce cytokines IL-4, IL-21, and IFN-γ which activate B cells that are capable of producing antibodies (16). Thus, cDC1 contributes to the formation of the humoral immune system.

Conventional dendritic cell 2 (cDC2) is a DC that has a broader cross-presentation capability to CD4+ and CD8+ T cells compared to other DCs (82). This DC is the leading producer of IL-1β, IL-6, IL-12, and IL-23 that makes DC as the most potent activator of Th1, Th2, and Th17 (73). The produced IL-12 is capable of regulating Transcription factor 1 (Tcf1) which is a regulator for the differentiation of CD8+ into effector cells as well as memory cells (78). Based on research, cDC2 is also an efficient Tfh inducer, thus making these cells have an essential role in antibody generation (16). In addition, cDC2 also plays a role in Tregs differentiation through the IL-10 and Transforming Growth Factor-β (TGF-β) production (72).

Monocyte derived dendritic cell (moDC) originate from monocytes during infection and inflammation (73). *In vitro*, moDC can be formed by administering Granulocyte-Macrophage Colony-Stimulating Facto*r* (GM-CSF) and IL-4 stimulation through the IRF-4 signaling pathway (75). Like other types of DCs, moDC has the ability to prime T cells through T-bet and Tcf1 regulation in line with the production of cytokines IL-1, IL-23, and TNF-α (72, 79). moDC also produces IL-15 causes memory CD8+ T cells last a long time (80). In addition, moDC also secretes IL-12 which can activate T cells that become Th1 cells (71).

## Rationale of dendritic cell based vaccine for SARS-CoV-2 infection

Dendritic cells have been widely developed and researched as immunotherapy in managing various diseases. DC-based immunotherapy has been tested on breast, prostate, melanoma, kidney, glioblastoma, ovarian, and lung cancers (83). Clinical trial studies of DC-based vaccines arrayed promising results, with a marked rise in the count of anti-tumor-specific CD8+ T cells (84). As an example, clinical trials in patients with advanced ovarian cancer given autologous DC vaccines pulsed with HOCl-oxidized tumor lysate (OC-DC) showed an increase in T cell response and a lengthening of the survival rate for two years to 100% accompanied by low side effects (85).

DC-based immunotherapy was also developed for infectious diseases. In HIV trials, DC-based vaccines increased specific T cells response, although the effectiveness of reducing viral load was still not conclusive (86). Clinical trials for hepatitis C also showed an upsurge of specific cellular immunity to HCV in the absence of severe side effects (87). Further, DC-based vaccines were also developed for hepatitis B, malaria, as well as influenza (11, 88, 89).

The success of DC-based cancer immunotherapy and infection vaccines suggests the potential for DC development as a SARS-CoV-2 vaccine. This approach utilizes the ability to present antigens and induce the immune system possessed by DC (90). Immature DCs can be introduced with SARS-CoV-2 antigens, for example, S protein which has proven to elicit an immune response (91). This process can be developed both *in-vivo* and *ex-vivo*, but the *ex-vivo* approach can be an option in developing this vaccine because of its feasibility and shortening of the processes that should occur in the body (92). The DCs that have been exposed to the antigen will undergo maturation and drain to the lymphoid organs, then present the antigen to the naïve T cells so that specific immunity to SARS-CoV-2 is formed (71). This approach is currently being developed in Indonesia and commonly known as Nusantara Vaccine.

**Figure 3:**
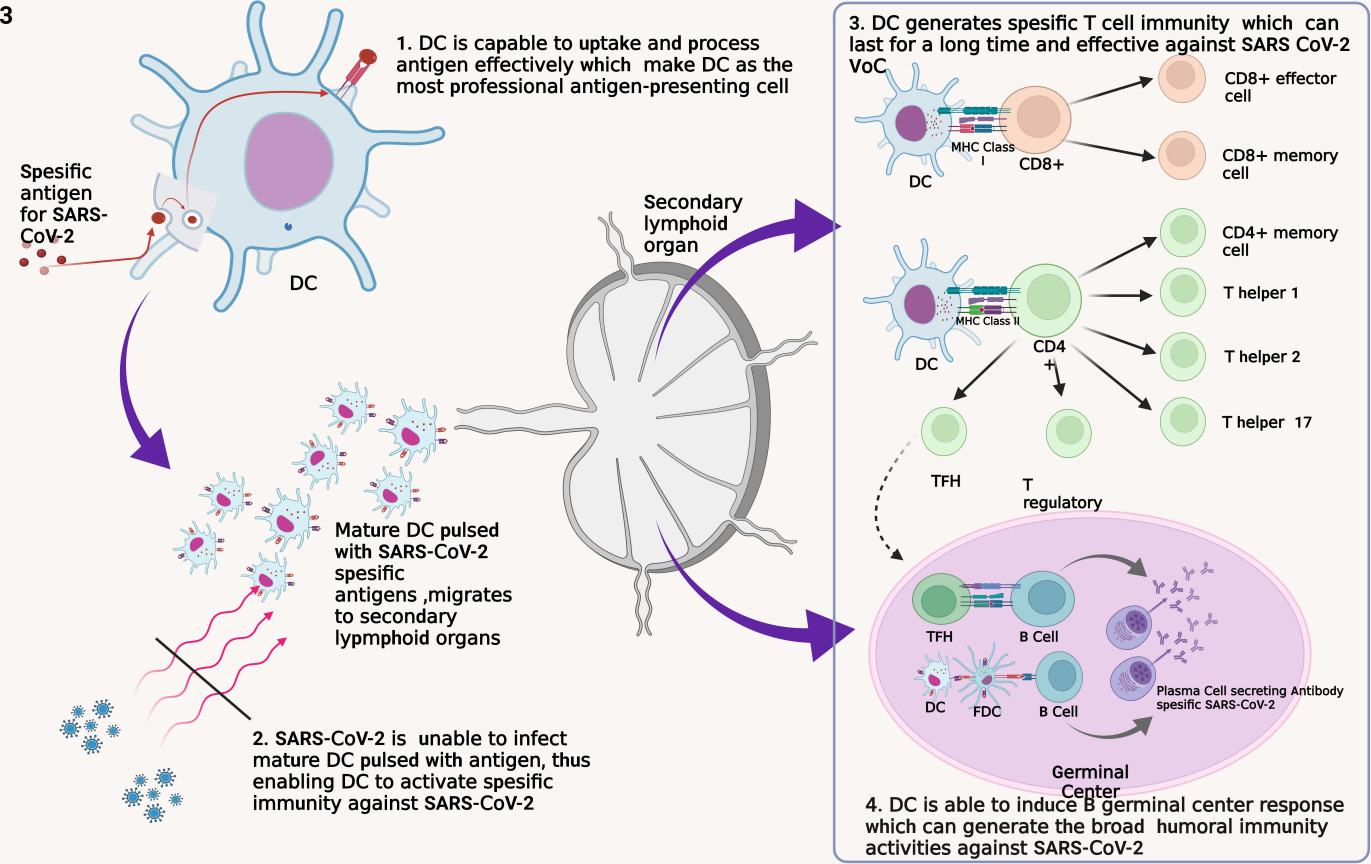
Four fundamental reasons for the development of DC as a SARS-CoV-2 vaccine. DC, dendritic cell; FDC, follicular dendritic cell; MHC, major histocompatibility complex; TFH, T follicular helper.

There are four main reasons that can support the utilization of DC as a SARS-CoV-2 vaccine, including (**Figure 3**):

First, DC is a professional APC that captures, processes, and exposes antigens efficiently and effectively to other immune cells such as T cells (32). DC recognizes and internalizes antigens by endocytosis or by direct contact with gap junction and by cross-dressing (93). This method allows the DC to be able to identify and capture antigens in other infected cells and those that have experienced apoptotic. DC has a lower lysosome protease enzyme and the ability to neutralize pH well to maintain the antigens captured until the exposure process to other immune cells (32). In addition, DC has Gamma Interferon-Induce Lysosomal Thiolreductase (GILT), whose function is to maintain intracellular processes in the DC so that pyroptotic does not occur due to inflammasome activation (94). Thus, the use of DC as a vaccine will ensure the process of introduction and presentation of SARS-CoV-2 antigens so that specific immunity formation occurs.

Second, DC is a cell that SARS-CoV-2 weakens to evade the body’s immune response thus, DC is a plausible vaccination target (95). In the acute phase, the significant decrease of pDC leads to IFN-I depletion thus, causes a failure of the innate immune response (96). In addition, SARS-CoV-2 infection also inhibits adaptive immune responses through impairing DC maturation characterized by a decrease in Human Leucocyte Antigen – DR isotype (HLA-DR) and CD80 expressions (49, 50). In COVID-19 patients, it was found that the reduction of DC was correlated with the depletion of T cell numbers (97). Altogether, this condition leads to the failure to transition an innate immune response into an adaptive immune response. Therefore, vaccination with a focus on improving and protecting DC function has the potential to provide better results.

Third, DC has a good ability for T cell activation. As previously explained, DC will activate various types of T cells. Naïve CD8+ T cells will be activated into effector and memory T cells (76). Formed Th2 and Tfh cells play a role in the differentiation of B cells into antibody-producing cells, while Treg cells control the function of other lymphocytes (75). Evidence that formed SARS-CoV-2-specific memory T cells persist for an extended period implies this vaccine can prevent infection and replication of SARS-CoV-2 in the long term (53, 60). In addition, studies have shown that memory T cells remain effective against VoC thus, this DC-based vaccine has the potential to persist effective against various mutated virus variants (35). All of these things are also supported by studies that show that T cells play an essential role in SARS-CoV-2 infection. Therefore, the ability of DC to activate T cells is the basis of the use of DC for SARS-CoV-2 vaccines that potential to have good effectiveness.

Fourth, the DC-based vaccine has the potential to trigger the formation of germinal center (GC) cell responses so that B cells are formed and can recognize virus variants. DC induces the response of B GC cells through the activation of naïve T cells into Tfh cells, which will then activate B cells (98, 99). The activation process triggers the formation of plasma and memory B cells that undergo affinity maturation and clonal evolution so that a broad B cell response is formed to fight viruses with an immense mutation rate such as SARS-CoV-2 (100). Through this mechanism, antibodies that can neutralize SARS-CoV-2 widely will be generated so that they are effective against various virus variants.

For these four reasons, DC can be used as a SARS CoV-2 vaccine. The immunity generated through this approach is oriented towards forming T cells so that the vaccine can last a long time and remain effective against the developing variants of SARS-CoV-2. DC-based vaccines also have the potential to create antibodies that have a broad response. The integration in producing specific T cells and antibodies is the main key to developing DC as a potential SARS-CoV-2 vaccine. For this reason, further studies need to be executed to prove the safety and effectiveness of DC-based vaccines.

## Translation of DC-based vaccine for SARS-CoV-2: Challenges and future perspective

DC-based vaccine translation depends on various factors, DC type selection and processing, antigen loading selection, and administration methods of DC-based vaccines (101). As already mentioned above, there are various subtypes of DC present in the human body. pDC is often associated and fights an important immunity protection to viral infections (96). cDC that is able to activate T cells widely so that it is postulated is able to activate CD4+ which plays a role in the formation of antibodies (77). However, the utilization of both subsets requires a more invasive procedure, and its proportion in the body <1% in the blood becomes an obstacle in its utilization (102). moDC is a DC subtype that is widely chosen because it is easily accessible from peripheral blood which is then incubated with GM-CSF and IL-4 (103). Vaccines using moDC in cancer have been shown to be able to form T cell immunity. However, some studies have shown the potential for moDC inferiority in priming T cells compared to cDC and pDC (104). However, some studies have also shown that the ability of antigen transfer or cross-presentation that moDC then introduce antigens to endogenous cDC in the body so that it is able to produce cytokines (IL-12) that are able to priming CD4 cells (105). As well as a study also showed the cancer DC vaccine that the activation ability of CD8+ is also spaced by endogenous DC interacting with the DC vaccine (106). Thus moDC is potential candidate in the development of a vaccine for SARS-CoV-2.

Selection of loaded-proteins or antigens is also critical issue since the protein is determinator of a specific immune response. In this case, the selection of specific proteins capable of triggering a strong immune response to SARS-CoV-2 must be determined properly because it is related to its effectiveness even against virus variants that continue to develop. Currently the S-protein is widely used as a target in vaccine development. Utilization of this protein includes the use of full-length SARS-CoV-2 S-proteins, specific sub-units of S-protein (S1, S2), and specific RBD S-protein SARS-CoV-2. S-protein plays important role in the entry of viruses, and several loci of its RBD are targets of the SARS-CoV-2 immunoglobulin antibody (107). This is supported by the results of research that S-protein is able to trigger specific immunity to SARS-CoV-2 (108). However, evidence also shows the occurrence of mutations in some loci in the S-protein which results in a decrease in the effectiveness of various other vaccines where there is a decrease in the affinity of the antibodies produced (109, 110).

The S-protein can still be an option in the development of DC-based vaccines. Given that DC-based vaccines are oriented towards the formation of T Cell immunity. As outlined, that the SARS-CoV-2 variant retains most of its epitope, specific research into epitope in the delta and omicron variants also shows that both variants still retain T cell epitopes by 75-90% (111). Therefore, the utilization of the S protein as a loaded-antigen in DC-based vaccines has the potential to maintain the effectiveness of the vaccine against the evolving SARS-CoV-2 variant according to the orientation of DC-based vaccines is the formation of T cells immunity. Determination of loaded-antigens in DC-based vaccines remains an opportunity for the foreseeable future. Determination of loaded-antigens in addition to affecting effectiveness, can also affect the load and cost of vaccine production.

## Conclusion

The development and discovery of effective and enduring vaccines remain a challenge in conquering the COVID-19 pandemic. Although various types of vaccines have been distributed, these vaccines still have limitations. The known professional ability of DC in activating T cells and their involvement in SARS-CoV-2 infection encourage the development of DC-based vaccines that have the potential to have good effectiveness. However, more research is still needed to get a safe and effective DC-based vaccine so that in the end it can be a breakthrough to overcome the ongoing pandemic.

## Author contributions

All authors contributed equally in concepting, drafting and revising the manuscript.

## Funding

This paper was funded by Cellcure Center Gatot Soebroto Central Army Hospital, Indonesia.

## Conflict of interest

The authors declare that the research was conducted in the absence of any commercial or financial relationships that could be construed as a potential conflict of interest.

## Publisher’s note

All claims expressed in this article are solely those of the authors and do not necessarily represent those of their affiliated organizations, or those of the publisher, the editors and the reviewers. Any product that may be evaluated in this article, or claim that may be made by its manufacturer, is not guaranteed or endorsed by the publisher.

## References

[1] Lu R Zhao X Li J Niu P Yang B Wu H. Genomic characterisation and epidemiology of 2019 novel coronavirus: Implications for virus origins and receptor binding. Lancet (2020) 395:565–74. doi: 10.1016/S0140-6736(20)30251-8 PMC7159086 32007145

[2] Andersen KG Rambaut A Lipkin WI Holmes EC Garry RF. The proximal origin of SARS-CoV-2. Nat Med (2020) 26:450–2. doi: 10.1038/s41591-020-0820-9 PMC7095063 32284615

[3] Gralinski L Menachery V. Return of the coronavirus: 2019-nCoV. Viruses (2020) 12:1–8. doi: 10.3390/v12020135 PMC7077245 31991541

[4] Malik JA Ahmed S Mir A Shinde M Bender O Alshammari F. The SARS-CoV-2 mutations versus vaccine effectiveness: New opportunities to new challenges. J Infect Public Health (2022) 15:228–40. doi: 10.1016/j.jiph.2021.12.014 PMC8730674 35042059

[5] Prompetchara E Ketloy C Palaga T. Immune responses in COVID-19 and potential vaccines: Lessons learned from SARS and MERS epidemic. Asian Pacific J Allergy Immunol (2020) 38:1–9. doi: 10.12932/AP-200220-0772 32105090

[6] Yang L Liu S Liu J Zhang Z Wan X Huang B. COVID-19: immunopathogenesis and immunotherapeutics. Signal Transduct Target Ther (2020) 5:1–8. doi: 10.1038/s41392-020-00243-2 32712629 PMC7381863

[7] Altmann DM Boyton RJ. SARS-CoV-2 T cell immunity: Specificity, function, durability, and role in protection. Sci Immunol (2020) 5:2–7. doi: 10.1126/sciimmunol.abd6160 32680954

[8] Fiolet T Kherabi Y MacDonald CJ Ghosn J Peiffer-Smadja N. Comparing COVID-19 vaccines for their characteristics, efficacy and effectiveness against SARS-CoV-2 and variants of concern: a narrative review. Clin Microbiol Infect (2022) 28:202–21. doi: 10.1016/j.cmi.2021.10.005 PMC8548286 34715347

[9] Jung JH Rha MS Sa M Choi HK Jeon JH Seok H. SARS-CoV-2-specific T cell memory is sustained in COVID-19 convalescent patients for 10 months with successful development of stem cell-like memory T cells. Nat Commun (2021) 12:1–12. doi: 10.1038/s41467-021-24377-1 34193870 PMC8245549

[10] Al-Ashmawy GMZ. “Dendritic cell subsets, maturation and function.,”. In: Chapoval SP, editor. Dendritic cells. London: IntechOpen. (2018). p. 11–24. doi: 10.5772/intechopen.79926

[11] Luo K Gordy JT Zavala F Markham RB. A chemokine-fusion vaccine targeting immature dendritic cells elicits elevated antibody responses to malaria sporozoites in infant macaques. Sci Rep (2021) 11:1–14. doi: 10.1038/s41598-020-79427-3 33441615 PMC7807052

[12] Zhao C Zhao W. NLRP3 inflammasome–a key player in antiviral responses. Front Immunol (2020) 11:211. doi: 10.3389/fimmu.2020.00211 32133002 PMC7040071

[13] García LF. Immune response, inflammation, and the clinical spectrum of COVID-19. Front Immunol (2020) 11:1441. doi: 10.3389/fimmu.2020.01441 32612615 PMC7308593

[14] Marshall JS Warrington R Watson W Kim HL. An introduction to immunology and immunopathology. Allergy Asthma Clin Immunol (2018) 14:5–14. doi: 10.1186/s13223-018-0278-1 PMC6156898 30263032

[15] Gaudino SJ Kumar P. Cross-talk between antigen presenting cells and T cells impacts intestinal homeostasis, bacterial infections, and tumorigenesis. Front Immunol (2019) 10:360. doi: 10.3389/fimmu.2019.00360 30894857 PMC6414782

[16] Tesfaye DY Gudjonsson A Bogen B Fossum E. Targeting conventional dendritic cells to fine-tune antibody responses. Front Immunol (2019) 10:1529. doi: 10.3389/fimmu.2019.01529 31333661 PMC6620736

[17] Laing AG Lorenc A del Molino del Barrio I Das A Fish M Monin L. A dynamic COVID-19 immune signature includes associations with poor prognosis. Nat Med (2020) 26:1623–35. doi: 10.1038/s41591-020-1038-6 32807934

[18] Huang C Wang Y Li X Ren L Zhao J Hu Y. Clinical features of patients infected with 2019 novel coronavirus in wuhan, China. Lancet (2020) 395:497–506. doi: 10.1016/S0140-6736(20)30183-5 31986264 PMC7159299

[19] Hu B Guo H Zhou P Shi ZL. Characteristics of SARS-CoV-2 and COVID-19. Nat Rev Microbiol (2021) 19:141–54. doi: 10.1038/s41579-020-00459-7 PMC7537588 33024307

[20] Chang FY Chen HC Chen PJ Ho MS Hsieh SL Lin JC. Immunologic aspects of characteristics, diagnosis, and treatment of coronavirus disease 2019 (COVID-19). J BioMed Sci (2020) 27:1–13. doi: 10.1186/s12929-020-00663-w PMC7270518 32498686

[21] Park A Iwasaki A. Type I and type III interferons – induction, signaling, evasion, and application to combat COVID-19. Cell Host Microbe (2020) 27:870–8. doi: 10.1016/j.chom.2020.05.008 PMC7255347 32464097

[22] Rosa BA Ahmed M Singh DK Choreño-Parra JA Cole J Jiménez-Álvarez LA. IFN signaling and neutrophil degranulation transcriptional signatures are induced during SARS-CoV-2 infection. Commun Biol (2021) 4:1–14. doi: 10.1038/s42003-021-01829-4 33674719 PMC7935909

[23] Ivashkiv LB Donlin LT. Regulation of type I interferon responses. Nat Rev Immunol (2014) 14:36–49. doi: 10.1038/nri3581 24362405 PMC4084561

[24] Vabret N Britton GJ Gruber C Hegde S Kim J Kuksin M. Immunology of COVID-19: Current state of the science. Immunity (2020) 52:910–41. doi: 10.1016/j.immuni.2020.05.002 PMC7200337 32505227

[25] Lei X Dong X Ma R Wang W Xiao X Tian Z. Activation and evasion of type I interferon responses by SARS-CoV-2. Nat Commun (2020) 11:1–12. doi: 10.1038/s41467-020-17665-9 32733001 PMC7392898

[26] Taefehshokr N Taefehshokr S Hemmat N Heit B. Covid-19: Perspectives on innate immune evasion. Front Immunol (2020) 11:580641. doi: 10.3389/fimmu.2020.580641 33101306 PMC7554241

[27] Pan P Shen M Yu Z Ge W Chen K Tian M. SARS-CoV-2 n protein promotes NLRP3 inflammasome activation to induce hyperinflammation. Nat Commun (2021) 12:1–17. doi: 10.1038/s41467-021-25015-6 34341353 PMC8329225

[28] Freeman TL Swartz TH. Targeting the NLRP3 inflammasome in severe COVID-19. Front Immunol (2020) 11:1518. doi: 10.3389/fimmu.2020.01518 32655582 PMC7324760

[29] van den Berg DF te Velde AA. Severe COVID-19: NLRP3 inflammasome dysregulated. Front Immunol (2020) 11:1580. doi: 10.3389/fimmu.2020.01580 32670297 PMC7332883

[30] Tay MZ Poh CM Rénia L MacAry PA Ng LFP. The trinity of COVID-19: immunity, inflammation and intervention. Nat Rev Immunol (2020) 20:363–74. doi: 10.1038/s41577-020-0311-8 PMC7187672 32346093

[31] Forthal D. Adaptive immune responses to SARS-CoV-2. Adv Drug Delivery Rev (2021) 172:1–8. doi: 10.1016/j.addr.2021.02.009 PMC7891074 33610693

[32] Embgenbroich M Burgdorf S. Current concepts of antigen cross-presentation. Front Immunol (2018) 9:1643. doi: 10.3389/fimmu.2018.01643 30061897 PMC6054923

[33] Grifoni A Weiskopf D Ramirez SI Mateus J Dan JM Moderbacher CR. Targets of T cell responses to SARS-CoV-2 coronavirus in humans with COVID-19 disease and unexposed individuals. Cell (2020) 181:1489–501. doi: 10.1016/j.cell.2020.05.015 PMC7237901 32473127

[34] Grifoni A Sidney J Vita R Peters B Crotty S Weiskopf D. SARS-CoV-2 human T cell epitopes : Adaptive immune response against COVID-19. Cell Host Microbe (2021) 29:1076–92. doi: 10.1016/j.chom.2021.05.010 PMC8139264 34237248

[35] Moss P. The T cell immune response against SARS-CoV-2. Nat Immunol (2022) 23:186–93. doi: 10.1038/s41590-021-01122-w 35105982

[36] Tan AT Linster M Tan CW Le Bert N Chia WN Kunasegaran K. Early induction of functional SARS-CoV-2-specific T cells associates with rapid viral clearance and mild disease in COVID-19 patients. Cell Rep (2021) 34:108728. doi: 10.1016/j.celrep.2021.108728 33516277 PMC7826084

[37] Rha MS Shin EC. Activation or exhaustion of CD8+ T cells in patients with COVID-19. Cell Mol Immunol (2021) 18:2325–33. doi: 10.1038/s41423-021-00750-4 PMC8374113 34413488

[38] Zheng HY Zhang M Yang CX Zhang N Wang XC Yang XP. Elevated exhaustion levels and reduced functional diversity of T cells in peripheral blood may predict severe progression in COVID-19 patients. Cell Mol Immunol (2020) 17:541–3. doi: 10.1038/s41423-020-0401-3 PMC7091621 32203186

[39] Wang F Nie J Wang H Zhao Q Xiong Y Deng L. Characteristics of peripheral lymphocyte subset alteration in covid-19 pneumonia. J Infect Dis (2020) 221:1762–9. doi: 10.1093/INFDIS/JIAA150 PMC7184346 32227123

[40] Xiang Q Feng Z Diao B Tu C Qiao Q Yang H. SARS-CoV-2 induces lymphocytopenia by promoting inflammation and decimates secondary lymphoid organs. Front Immunol (2021) 12:661052. doi: 10.3389/fimmu.2021.661052 33995382 PMC8113960

[41] André S Picard M Cezar R Roux-Dalvai F Alleaume-Butaux A Soundaramourty C. T Cell apoptosis characterizes severe covid-19 disease. Cell Death Differ (2022) 29:1–14. doi: 10.1038/s41418-022-00936-x 35066575 PMC8782710

[42] Liu Y Tan W Chen H Zhu Y Wan L Jiang K. Dynamic changes in lymphocyte subsets and parallel cytokine levels in patients with severe and critical COVID-19. BMC Infect Dis (2021) 21:1–10. doi: 10.1186/s12879-021-05792-7 33461503 PMC7812569

[43] Saichi M Ladjemi MZ Korniotis S Rousseau C Ait Hamou Z Massenet-Regad L. Single-cell RNA sequencing of blood antigen-presenting cells in severe COVID-19 reveals multi-process defects in antiviral immunity. Nat Cell Biol (2021) 23:538–51. doi: 10.1038/s41556-021-00681-2 33972731

[44] Yang D Chu H Hou Y Chai Y Shuai H Lee ACY. Attenuated interferon and proinflammatory response in SARS-CoV-2-infected human dendritic cells is associated with viral antagonism of STAT1 phosphorylation. J Infect Dis (2020) 222:734–45. doi: 10.1093/infdis/jiaa356 PMC7337793 32563187

[45] Zhou R To KKW Wong YC Liu L Zhou B Li X. Acute SARS-CoV-2 infection impairs dendritic cell and T cell responses. Immunity (2020) 53:864–877.e5. doi: 10.1016/j.immuni.2020.07.026 32791036 PMC7402670

[46] de Cevins C Luka M Smith N Meynier S Magérus A Carbone F. A monocyte/dendritic cell molecular signature of SARS-CoV-2-related multisystem inflammatory syndrome in children with severe myocarditis. Med (2021) 2:1072–1092.e7. doi: 10.1016/j.medj.2021.08.002 34414385 PMC8363470

[47] Pérez-Gómez A Vitallé J Gasca-Capote C Gutierrez-Valencia A Trujillo-Rodriguez M Serna-Gallego A. Dendritic cell deficiencies persist seven months after SARS-CoV-2 infection. Cell Mol Immunol (2021) 18:2128–39. doi: 10.1038/s41423-021-00728-2 PMC8294321 34290398

[48] Borcherding L Teksen AS Grosser B Schaller T Hirschbühl K Claus R. Impaired dendritic cell homing in COVID-19. Front Med (2021) 8:761372. doi: 10.3389/fmed.2021.761372 PMC8601231 34805226

[49] Silvin A Chapuis N Dunsmore G Goubet AG Dubuisson A Derosa L. Elevated calprotectin and abnormal myeloid cell subsets discriminate severe from mild COVID-19. Cell (2020) 182:1401–1418.e18. doi: 10.1016/j.cell.2020.08.002 32810439 PMC7405878

[50] Schulte-Schrepping J Reusch N Paclik D Baßler K Schlickeiser S Zhang B. Severe COVID-19 is marked by a dysregulated myeloid cell compartment. Cell (2020) 182:1419–1440.e23. doi: 10.1016/j.cell.2020.08.001 32810438 PMC7405822

[51] Galati D Zanotta S Capitelli L Bocchino M. A bird’s eye view on the role of dendritic cells in SARS-CoV-2 infection: Perspectives for immune-based vaccines. Allergy Eur J Allergy Clin Immunol (2022) 77:100–10. doi: 10.1111/all.15004 PMC8441836 34245591

[52] Long QX Liu BZ Deng HJ Wu GC Deng K Chen YK. Antibody responses to SARS-CoV-2 in patients with COVID-19. Nat Med (2020) 26:845–8. doi: 10.1038/s41591-020-0897-1 32350462

[53] Gurevich M Zilkha-Falb R Sonis P Magalashvili D Menascu S Flechter S. SARS-CoV-2 memory b and T cell profiles in mild COVID-19 convalescent patients. Int J Infect Dis (2022) 115:208–14. doi: 10.1016/j.ijid.2021.12.309 PMC8653411 34896265

[54] Sekine T Perez-Potti A Rivera-Ballesteros O Strålin K Gorin JB Olsson A. Robust T cell immunity in convalescent individuals with asymptomatic or mild COVID-19. Cell (2020) 183:158–168.e14. doi: 10.1016/j.cell.2020.08.017 32979941 PMC7427556

[55] Löfström E Eringfält A Kötz A Wickbom F Tham J Lingman M. Dynamics of IgG-avidity and antibody levels after covid-19. J Clin Virol (2021) 144:1–6. doi: 10.1016/j.jcv.2021.104986 PMC8451979 34563862

[56] Xiang T Liang B Fang Y Lu S Li S Wang H. Declining levels of neutralizing antibodies against SARS-CoV-2 in convalescent COVID-19 patients one year post symptom onset. Front Immunol (2021) 12:708523. doi: 10.3389/fimmu.2021.708523 34220870 PMC8242354

[57] Chen Z John Wherry E. T Cell responses in patients with COVID-19. Nat Rev Immunol (2020) 20:529–36. doi: 10.1038/s41577-020-0402-6 PMC7389156 32728222

[58] Notarbartolo S Ranzani V Bandera A Gruarin P Bevilacqua V Putignano AR. Integrated longitudinal immunophenotypic, transcriptional and repertoire analyses delineate immune responses in COVID-19 patients. Sci Immunol (2021) 6:1–19. doi: 10.1126/sciimmunol.abg5021 34376481

[59] Peng Y Mentzer AJ Liu G Yao X Yin Z Dong D. Broad and strong memory CD4+ and CD8+ T cells induced by SARS-CoV-2 in UK convalescent individuals following COVID-19. Nat Immunol (2020) 21:1336–45. doi: 10.1038/s41590-020-0782-6 PMC7611020 32887977

[60] Adamo S Michler J Zurbuchen Y Cervia C Taeschler P Raeber ME. Signature of long-lived memory CD8+ T cells in acute SARS-CoV-2 infection. Nature (2021), 148–55. doi: 10.1038/s41586-021-04280-x PMC8810382 34875673

[61] Bertoletti A Le Bert N Qui M Tan AT. SARS-CoV-2-specific T cells in infection and vaccination. Cell Mol Immunol (2021) 18:2307–12. doi: 10.1038/s41423-021-00743-3 PMC8408362 34471260

[62] Breton G Mendoza P Hägglöf T Oliveira TY Schaefer-Babajew D Gaebler C. Persistent cellular immunity to SARS-CoV-2 infection. J Exp Med (2021) 218:1–11. doi: 10.1084/JEM.20202515 PMC7845919 33533915

[63] Noh JY Jeong HW Kim JH Shin EC. T Cell-oriented strategies for controlling the COVID-19 pandemic. Nat Rev Immunol (2021) 21:687–8. doi: 10.1038/s41577-021-00625-9 PMC8424399 34497383

[64] Menni C Klaser K May A Polidori L Capdevila J Louca P. Vaccine side-effects and SARS-CoV-2 infection after vaccination in users of the COVID symptom study app in the UK: a prospective observational study. Lancet Infect Dis (2021) 21:939–49. doi: 10.1016/S1473-3099(21)00224-3 PMC8078878 33930320

[65] Baden LR El Sahly HM Essink B Kotloff K Frey S Novak R. Efficacy and safety of the mRNA-1273 SARS-CoV-2 vaccine. N Engl J Med (2021) 384:403–16. doi: 10.1056/nejmoa2035389 PMC7787219 33378609

[66] Logunov DY Dolzhikova IV Zubkova OV Tukhvatullin AI Shcheblyakov DV Dzharullaeva AS. Safety and immunogenicity of an rAd26 and rAd5 vector-based heterologous prime-boost COVID-19 vaccine in two formulations: two open, non-randomised phase 1/2 studies from Russia. Lancet (2020) 396:887–97. doi: 10.1016/S0140-6736(20)31866-3 PMC7471804 32896291

[67] Voysey M Clemens SAC Madhi SA Weckx LY Folegatti PM Aley PK. Safety and efficacy of the ChAdOx1 nCoV-19 vaccine (AZD1222) against SARS-CoV-2: an interim analysis of four randomised controlled trials in Brazil, south Africa, and the UK. Lancet (2021) 397:99–111. doi: 10.1016/S0140-6736(20)32661-1 33306989 PMC7723445

[68] Zhang Y Zeng G Pan H Li C Hu Y Chu K. Safety, tolerability, and immunogenicity of an inactivated SARS-CoV-2 vaccine in healthy adults aged 18–59 years: a randomised, double-blind, placebo-controlled, phase 1/2 clinical trial. Lancet Infect Dis (2021) 21:181–92. doi: 10.1016/S1473-3099(20)30843-4 PMC7832443 33217362

[69] Dunkle LM Kotloff KL Gay CL Áñez G Adelglass JM Barrat Hernández AQ. Efficacy and safety of NVX-CoV2373 in adults in the united states and Mexico. N Engl J Med (2022) 386:531–43. doi: 10.1056/nejmoa2116185 PMC8693692 34910859

[70] Enamorado M Khouili SC Iborra S Sancho D. Genealogy, dendritic cell priming, and differentiation of tissue-resident memory CD8+ T cells. Front Immunol (2018) 9:1751. doi: 10.3389/fimmu.2018.01751 30108585 PMC6079237

[71] Liu J Zhang X Cheng Y Cao X. Dendritic cell migration in inflammation and immunity. Cell Mol Immunol (2021) 18:2461–71. doi: 10.1038/s41423-021-00726-4 PMC8298985 34302064

[72] Collin M Bigley V. Human dendritic cell subsets: an update. Immunology (2018) 154:3–20. doi: 10.1111/imm.12888 29313948 PMC5904714

[73] Balan S Saxena M Bhardwaj N. “Dendritic cell subsets and locations.,”. In: Lhuillier C Galluzzi L, editors. International review of cell and molecular biology. Elsevier Inc (2019). p. 1–68. doi: 10.1016/bs.ircmb.2019.07.004 31810551

[74] Barnaba V. T Cell memory in infection, cancer, and autoimmunity. Front Immunol (2022) 12:811968. doi: 10.3389/fimmu.2021.811968 35069600 PMC8771143

[75] Hilligan KL Ronchese F. Antigen presentation by dendritic cells and their instruction of CD4+ T helper cell responses. Cell Mol Immunol (2020) 17:587–99. doi: 10.1038/s41423-020-0465-0 PMC7264306 32433540

[76] Fu C Peng P Loschko J Feng L Pham P Cui W. Plasmacytoid dendritic cells cross-prime naive CD8 T cells by transferring antigen to conventional dendritic cells through exosomes. Proc Natl Acad Sci U.S.A. (2020) 117:23730–41. doi: 10.1073/pnas.2002345117 PMC7519282 32879009

[77] Cance JC Crozat K Dalod M Mattiuz R. Are conventional type 1 dendritic cells critical for protective antitomor immunity and how? Front Immunol (2019) 10:9. doi: 10.3389/fimmu.2019.00009 30809220 PMC6379659

[78] Danilo M Chennupati V Silva JG Siegert S Held W. Suppression of Tcf1 by inflammatory cytokines facilitates effector CD8 T cell differentiation. Cell Rep (2018) 22:2107–17. doi: 10.1016/j.celrep.2018.01.072 29466737

[79] Shin KS Jeon I Kim BS Kim IK Park YJ Koh CH. Monocyte-derived dendritic cells dictate the memory differentiation of CD8+ T cells during acute infection. Front Immunol (2019) 10:1887. doi: 10.3389/fimmu.2019.01887 31474983 PMC6706816

[80] Chu K-L Batista NV Girard M Watts TH. Monocyte-derived cells in tissue-resident memory T cell formation. J Immunol (2020) 204:477–85. doi: 10.4049/jimmunol.1901046 31964721

[81] Greene TT Zuniga EI. Type I interferon induction and exhaustion during viral infection: Plasmacytoid dendritic cells and emerging COVID-10 findings. Viruses (2021) 13:1839. doi: 10.3390/v13091839 34578420 PMC8472174

[82] Patente TA Pinho MP Oliveira AA Evangelista GCM Bergami-Santos PC Barbuto JAM. Human dendritic cells: Their heterogeneity and clinical application potential in cancer immunotherapy. Front Immunol (2019) 10:3176. doi: 10.3389/fimmu.2018.03176 PMC6348254 30719026

[83] Saadeldin MK Abdel-Aziz AK Abdellatif A. Dendritic cell vaccine immunotherapy; the beginning of the end of cancer and COVID-19. A hypothesis. Med Hypotheses (2021) 146:1–7. doi: 10.1016/j.mehy.2020.110365 PMC7836805 33221134

[84] Mastelic-Gavillet B Balint K Boudousquie C Gannon PO Kandalaft LE. Personalized dendritic cell vaccines-recent breakthroughs and encouraging clinical results. Front Immunol (2019) 10:766. doi: 10.3389/fimmu.2019.00766 31031762 PMC6470191

[85] Tanyi JL Bobisse S Ophir E Tuyaerts S Roberti A Genolet R. Personalized cancer vaccine effectively mobilizes antitumor T cell immunity in ovarian cancer. Sci Transl Med (2018) 10:1–15. doi: 10.1126/scitranslmed.aao5931 29643231

[86] da Silva LT Santillo BT de Almeida A Duarte AJ da S Oshiro TM. Using dendritic cell-based immunotherapy to treat HIV: How can this strategy be improved? Front Immunol (2018) 9:2993. doi: 10.3389/fimmu.2018.02993 30619346 PMC6305438

[87] Zabaleta A D’Avola D Echeverria I Llopiz D Silva L Villanueva L. Clinical testing of a dendritic cell targeted therapeutic vaccine in patients with chronic hepatitis c virus infection. Mol Ther - Methods Clin Dev (2015) 2:15006. doi: 10.1038/mtm.2015.6 26029717 PMC4444996

[88] Ahn H Weaver M Lyon D Eunyoung C Roger B. Influenza vaccines differentially regulate the interferon response in human dendritic cells subset. Sci Transl Med (2017) 9:1–21. doi: 10.1126/scitranslmed.aaf9194.Influenza PMC5484150 28330867

[89] George R Ma A Motyka B Shi YE Liu Q Griebel P. And humoral immune responses. vivo. Hum Vaccines Immunother (2020) 16:779–92. doi: 10.1080/21645515.2019.1689081 PMC7227651 31687875

[90] Moticka EJ. Role of dendritic cells in the adaptive immune response. A Hist Perspect Evidence-Based Immunol (2016), 253–9. doi: 10.1016/b978-0-12-398381-7.00029-0

[91] Ravichandran S Coyle EM Klenow L Tang J Grubbs G Liu S. Antibody signature induced by SARS-CoV-2 spike protein immunogens in rabbits. Sci Transl Med (2020) 12:1–8. doi: 10.1126/SCITRANSLMED.ABC3539 PMC7286538 32513867

[92] Jonny Putranto TA Sitepu EC Irfon R. Dendritic cell vaccine as a potential strategy to end the COVID-19 pandemic. why should it be ex vivo? Expert Review of Vaccine, (2022) 21(8):1111–20. doi: 10.1080/14760584.2022.2080658. 35593184

[93] Campana S De Pasquale C Carrega P Ferlazzo G Bonaccorsi I. Cross-dressing: An alternative mechanism for antigen presentation. Immunol Lett (2015) 168:349–54. doi: 10.1016/j.imlet.2015.11.002 26551033

[94] Wagner CS Grotzke J Cresswell P. Intracellular regulation of cross-presentation during dendritic cell maturation. PloS One (2013) 8:1–13. doi: 10.1371/journal.pone.0076801 PMC3789698 24098562

[95] Borges RC Hohmann MS Borghi SM. Dendritic cells in COVID-19 immunopathogenesis: insights for a possible role in determining disease outcome. Int Rev Immunol (2021) 40:108–25. doi: 10.1080/08830185.2020.1844195 33191813

[96] Caldarale F Giacomelli M Garrafa E Tamassia N Morreale A Poli P. Plasmacytoid dendritic cells depletion and elevation of IFN-γ dependent chemokines CXCL9 and CXCL10 in children with multisystem inflammatory syndrome. Front Immunol (2021) 12:654587. doi: 10.3389/fimmu.2021.654587 33841438 PMC8033149

[97] Chang T Yang J Deng H Chen D Yang XP Tang ZH. Depletion and dysfunction of dendritic cells: Understanding SARS-CoV-2 infection. Front Immunol (2022) 13:843342. doi: 10.3389/fimmu.2022.843342 35265087 PMC8898834

[98] Koutsakos M Nguyen THO Kedzierska K. With a little help from T follicular helper friends: Humoral immunity to influenza vaccination. J Immunol (2019) 202:360–7. doi: 10.4049/jimmunol.1800986 30617117

[99] Heath WR Kato Y Steiner TM Caminschi I. Antigen presentation by dendritic cells for b cell activation. Curr Opin Immunol (2019) 58:44–52. doi: 10.1016/j.coi.2019.04.003 31071588

[100] Laidlaw BJ Ellebedy AH. The germinal centre b cell response to SARS-CoV-2. Nat Rev Immunol (2022) 22:7–18. doi: 10.1038/s41577-021-00657-1 34873279 PMC8647067

[101] Nazarkina ZK Zajakina A Laktionov PP. Maturation and antigen loading protocols influence activity of anticancer dendritic cells. Mol Biol (2018) 52:222–31. doi: 10.1134/S0026893317050132 29695694

[102] Macri C Pang ES Patton T O’Keeffe M. Dendritic cell subsets. Semin Cell Dev Biol (2018) 84:11–21. doi: 10.1016/j.semcdb.2017.12.009 29246859

[103] Zhuo G Zhao X Song X R. *Ex-vivo* pulsed dendritic cell vaccination against cancer. Acta Pharmacol Sin (2020) 41:959–69. doi: 10.1038/s41401-020-0415-5 PMC7470877 32366940

[104] Zhou Y Slone N Chrisikos TT Kyrysyuk O Babcock RL Medik YB. Vaccine efficacy against primary and metastatic cancer with *in vitro*-generated CD103 + conventional dendritic cells. J Immunother Cancer (2020) 8:1–13. doi: 10.1136/jitc-2019-000474 PMC7254126 32273347

[105] Ashour DED Arampatzi P Pavlovic V Förstner KU Kaisho T Beilhack A. IL-12 from endogenous cDC1, and not Is required for Th1 induction. JCI Insight (2020) 5:1–16. doi: 10.1172/JCI.INSIGHT.135143 PMC7259537 32434994

[106] Yewdall AW Drutman SB Jinwala F Bahjat KS Bhardwaj N. CD8+ T cell priming by dendritic cell vaccines requires antigen transfer to endogenous antigen presenting cells. PloS One (2010) 5:1–10. doi: 10.1371/journal.pone.0011144 PMC2886840 20585396

[107] Martínez-Flores D Zepeda-Cervantes J Cruz-Reséndiz A Aguirre-Sampieri S Sampieri A Vaca L. SARS-CoV-2 vaccines based on the spike glycoprotein and implications of new viral variants. Front Immunol (2021) 12:701501. doi: 10.3389/fimmu.2021.701501 34322129 PMC8311925

[108] Kyriakidis NC López-Cortés A González EV Grimaldos AB Prado EO. SARS-CoV-2 vaccines strategies: A comprehensive review of phase 3 candidates. NPJ Vaccines (2021) 6:1–17. doi: 10.1038/s41541-021-00292-w PMC7900244 33619260

[109] Alefishat E Jelinek HF Mousa M Tay GK Alsafar HS. Immune response to SARS-CoV-2 variants: A focus on severity, susceptibility, and preexisting immunity. J Infect Public Health (2022) 15:277–88. doi: 10.1016/j.jiph.2022.01.007 PMC8757655 35074728

[110] Geers D Shamier MC Bogers S den Hartog G Gommers L Nieuwkoop NN. SARS-CoV-2 variants of concern partially escape humoral but not T-cell responses in COVID-19 convalescent donors and vaccinees. Sci Immunol (2021) 6:1–15. doi: 10.1126/sciimmunol.abj1750 PMC9268159 34035118

[111] Sankaranarayanan S Mohkhedkar M Janakiraman V. Mutations in spike protein T cell epitopes of SARS-CoV-2 variants: Plausible influence on vaccine efficacy. Mol Basis Dis (2022) 1868:1–19. doi: 10.1016/j.bbadis.2022.166432 PMC9109158 35568352

